# Congenital and Acquired Chronic Neutropenias: Challenges, Perspectives and Implementation of the EuNet-INNOCHRON Action

**DOI:** 10.1097/HS9.0000000000000406

**Published:** 2020-06-08

**Authors:** Helen A. Papadaki, Irene Mavroudi, Antonio Almeida, Juergen Bux, Joanna Cichy, David C. Dale, Jean Donadieu, Petter Höglund, Oliver Karanfilski, Cristina Mecucci, Jan Palmblad, Julia Skokowa, Kostas Stamatopoulos, Ivo Touw, Alan J. Warren, Karl Welte, Cornelia Zeidler, Carlo Dufour

**Affiliations:** 1 Haemopoiesis Research Laboratory, School of Medicine, University of Crete; Department of Hematology, University Hospital of Heraklion, Crete, Greece; 2 Department of Hematology, Hospital da Luz Lisboa, Portugal; 3 Ruhr University, Bochum, Germany; 4 Department of Immunology, Faculty of Biochemistry, Biophysics and Biotechnology, Jagiellonian University, Kraków, Poland; 5 University of Washington, Seattle, WA, USA; 6 Department of Pediatric Hematology and Oncology, Trousseau Hospital, AP-HP, Paris, France; 7 Clinical Immunology and Transfusion Medicine Clinic, Karolinska University Hospital, Stockholm, Sweden; 8 Center for Hematology and Regenerative Medicine (HERM), Department of Medicine Huddinge, Karolinska Institutet, Stockholm, Sweden; 9 Hematology Clinic, Karolinska University Hospital, Stockholm, Sweden; 10 University Clinic for Hematology, Skopje, North Macedonia; 11 Hematology and Bone Marrow Transplantation Unit, Department of Medicine, Molecular Medicine Laboratory, University of Perugia, Perugia, Italy; 12 Department of Oncology, Hematology, Immunology, Rheumatology and Clinical Immunology, University Hospital Tübingen, Tübingen, Germany; 13 Institute of Applied Biosciences, Centre for Research and Technology Hellas, Thessaloniki, Greece; 14 Department of Hematology, Erasmus University Medical Centre, Rotterdam, Netherlands; 15 Department of Hematology, University of Cambridge; Cambridge Institute for Medical Research, University of Cambridge; Wellcome Trust–Medical Research Council Stem Cell Institute, University of Cambridge, Cambridge, UK; 16 University Children's Hospital Tübingen, Tübingen, Germany; 17 Department of Oncology, Hematology, Immunology and Bone Marrow Transplantation, Hannover Medical School, Germany; 18 Hematology-Oncology-HSCT Pole, G.Gaslini IRCCS Children Hospital, Genova, Italy.

## Introduction

Chronic Neutropenias (CNP) represent a wide spectrum of disorders ranging from mild to life-threatening, congenital or acquired diseases characterized by absolute neutrophil counts below 1.5 × 10^9^ /L for at least 3 months.^1^

Congenital Neutropenias comprise rare disorders of neutrophil production associated with mutations in more than 20 recognized genes but also with still unknown genetic aberrations, resulting in impaired neutrophil differentiation and/or survival, varying degree of propensity to Myelodysplastic Syndrome (MDS)/Acute Myeloid Leukemia (AML) and frequent dysfunction of non-hemopoietic organs.^2–6^ Unfolded protein responses, endoplasmic reticulum stress, elevated apoptosis, deregulated expression of transcription factors, abnormalities in secretory vesicles and mitochondrial metabolism, aberrations in ribosome biogenesis and assembly have been recognized as possible pathogenetic mechanisms.^2–5^ Congenital Neutropenias also include benign familial or ethnic variations associated with polymorphisms of known or yet unidentified genes with unknown frequency and pathophysiologic significance that need further investigation.^7,8^ Acquired CNP encompass diverse disease entities mediated through neutrophil-directed antibodies, cellular (NK- or T-cell) or cytokine dependent immune processes or via unknown pathogenetic mechanisms.^9–12^ These latter categories, also known as idiopathic CNP, include benign and uncomplicated forms of the disease but also pre-MDS cases, associated or not with clonal hemopoiesis, that need early recognition and close monitoring.^9–12^

The treatment of patients with CNP depends on the type and severity of the underlying disease. The management ranges from simple follow-up of uncomplicated cases, to systemic administration of granulocyte colony stimulating factor (G-CSF), antibiotics and immunomodulatory agents, to more drastic therapies with the potential to cure, that is, hemopoietic stem cell (HSC) transplantation or even to experimental gene-based interventions using CRISPR-Cas9 technology.^1,13–16^

### The challenges in CNP and the importance of networking

The diagnosis of congenital and acquired CNP remains a challenge with the underlying mechanisms still obscure in 30% to 50% of patients. Moreover, the variability in genotypic/phenotypic characteristics and natural history of these patients has thus far precluded defining common treatment patterns and guidance whereas the rarity of the underlying diseases has resulted in difficulties in recruiting patients for clinical trials. The collaboration between clinicians and scientists with special interest in these diseases as well as the establishment and interconnection of CNP patient Registries and Biobanks are essential prerequisites for the generation of a rich source of real-world data that will both facilitate the accurate diagnosis and contribute to a consensus on patient management; foster research on the pathogenetic/pathophysiologic mechanisms; and, give more opportunities for the development of novel and personalized treatment approaches as well as initiation of clinical trials.

**Figure 1:**
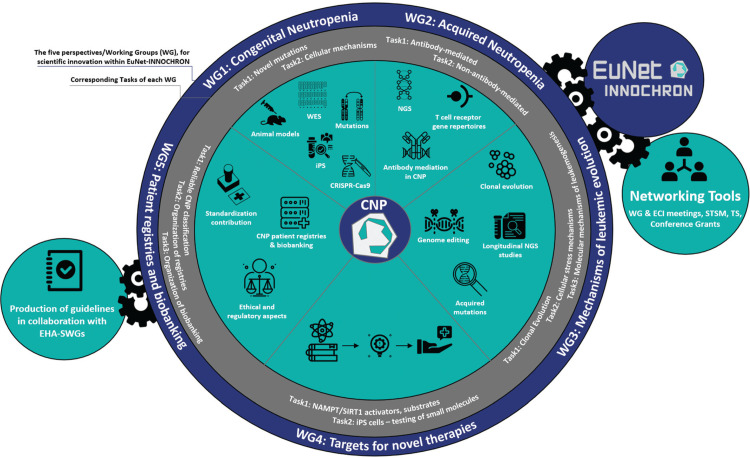
**A schematic representation of the EuNet-INNOCHRON Action.** The figure depicts the main WG of the Action with the respective Tasks as well as the tools for addressing the challenges in the field of CNP. The main networking tools to achieve the objectives of the Action are also shown. CNP = chronic neutropenia, ECI = early career investigators, EHA-SWG = European Hematology Association – Scientific Working Groups, NGS = next generation sequencing, STSM = short term scientific missions, TS = training schools, WES = whole exome sequencing, WG = working group.

A wide European network for the diagnosis and treatment of CNP (EuNet-INNOCHRON; https://www.eunet-innochron.eu/) funded by the European Cooperation of Science and Technology (COST) was launched in November 2019 (Fig. 1). It consists of clinicians and scientists from diverse scientific areas, early career investigators and biotechnological enterprises from more than 30 countries, mostly European, while also involving as collaborating partners the Severe Chronic Neutropenia International Registry (SCNIR; https://severe-chronic-neutropenia.org) and the European Hematology Association (EHA). EuNet-INNOCHRON is open to any clinician, researcher, scientific or patient society with a special focus in CNP. The network aims to: (a) harmonize the laboratory investigation of different types of CNP by facilitating the exchange of knowledge, tools, reagents, protocols and experience through inter-institutional collaborations; (b) formulate common diagnostic algorithms and treatment guidelines for patients with different types of CNPs aligned with the concepts of precision medicine; (c) organize new and expand existing CNP patient Registries and Biobanks using common protocols for sample collection, storage and management as well as template forms for patients’ informed consent according to the ethical standards of the European Legal Framework and the national and local regulations; (d) collect real-world data on the epidemiology, clinical presentation and natural course of CNPs and identify markers for improved decision-making and risk-adapted treatment strategies; (e) promote training and education of young investigators on advanced techniques; (f) foster entrepreneurial innovation and explore novel approaches for targeted drug development and innovative clinical trial design in collaboration with industrial partners. EuNet-INNOCHRON has adopted an excellence and inclusiveness policy and gives particular interest in the involvement of young researchers by offering a number of networking tools such as scientific meetings, short term scientific mission exchanges, training schools and conference grants.

### Perspectives for scientific innovations within EuNet-INNOCHRON

Five distinct but complementary Working Groups (WG) have been carefully designed within EuNet-INNOCHRON to address the challenges in CNP and promote breakthrough scientific developments and new concepts in the diagnosis, management and treatment of these patients (Table 1).

**Table 1:**
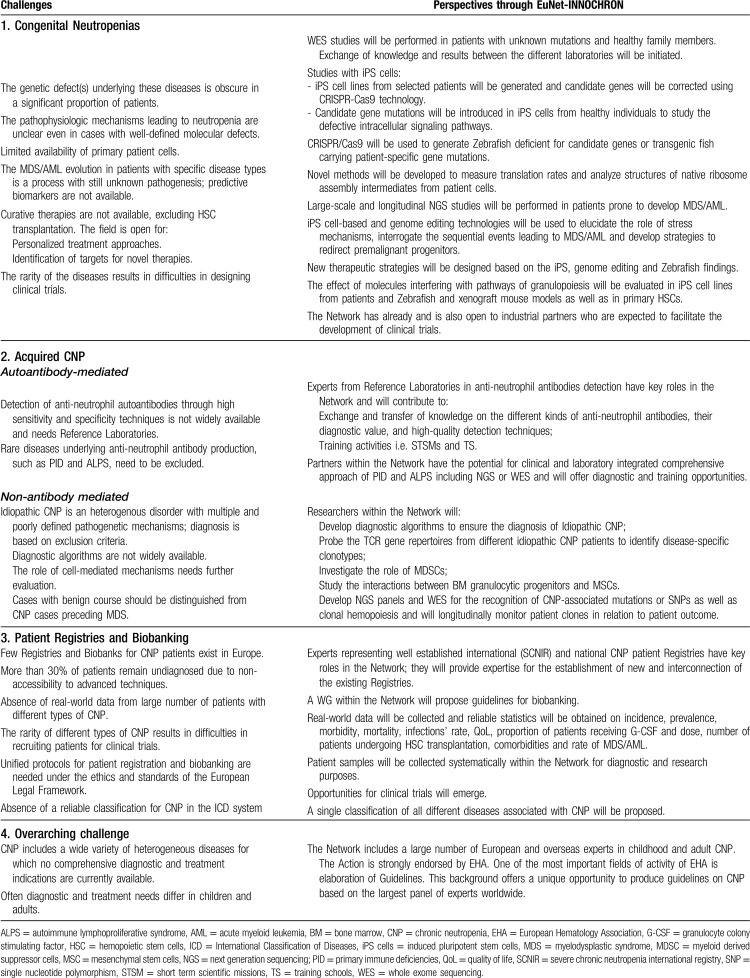
Challenges in CNP and perspectives through EuNet-INNOCHRON

### Congenital neutropenias

This WG will perform whole exome sequencing (WES) studies in patients with unknown mutations and healthy family members and promote collaborations in this field; candidate genes will be functionally interrogated for their role in granulocyte differentiation by generating induced pluripotent stem (iPS) cell lines from patients. Abnormal genes will be corrected in patient-derived iPS cells using CRISPR-Cas9 technology and the potential improvement of in vitro granulocytic differentiation of the gene corrected iPS cell lines will be evaluated. Candidate gene mutations will be also introduced into iPS cells derived from healthy individuals and their effects on granulocyte differentiation will be evaluated in vitro.^14,17^ RNA-sequencing analysis of HSCs generated from isogenic iPS cell lines will help to identify deregulated intracellular signaling pathways and candidate factors underlying the cellular phenotypes of neutropenias. CRISPR-Cas9 will be used to generate Zebrafish either deficient for the candidate genes or carrying patient-specific gene mutations. This WG will also study the CNP associated with Shwachman-Diamond Syndrome (SDS) to better understand the role of the causative genes *SBDS, EFL1,* and *DNAJC21* in ribosome assembly and also provide templates for the development of novel therapeutics.^18^ Robust, viable animal models will be generated and exploited to better understand SDS pathogenesis and promote the development of new therapeutics. A key objective of this WG is to establish new collaborations to achieve our goals.

### Acquired CNP

This WG will invest in collaborative work for the investigation of antibody- and non-antibody mediated CNP. An important goal is to raise awareness of the diversity of acquire CNP and the available diagnostic platforms. Emphasis will be given on the transfer of knowledge and methodological hands-on training on the high-quality detection of anti-neutrophil autoantibodies (sensitivity and specificity). Many traditional techniques are present in special laboratories whereas new diagnostic platforms are available with a potential for more wide-spread use. Novel diagnostic tools, such as next generation sequencing (NGS) including WES to understand better rare diseases that can by themselves cause antibody-mediated CNP, such as autoimmune lymphoproliferative syndrome (ALPS) and primary immune deficiencies (PID), will be performed.^19^ Regarding the non-antibody-mediated CNPs, particular networking will be directed to the idiopathic cases. Specifically, the WG will help to probe the T cell receptor gene repertoires from different patients with idiopathic CNP using NGS and results will be compared with public databases or databases from other diseases with overlapping features (eg, proliferations of T large granular lymphocytes, other bone marrow [BM] failure syndromes) to identify disease-specific but also shared clonotypes.^11^ The role of myeloid cell populations, namely myeloid derived suppressor cells (MDSC), will also be explored in idiopathic CNP as well as the significance of clonal hemopoiesis in correlation with patient outcome.^20^

### Mechanisms of leukemic evolution

MDS/AML evolution in specific types of CNPs is a multistep process of sequential hits and predictive biomarkers are not available.^21^ Congenital CNP is an interesting model to study leukemogenesis considering that the very first mutation of the process is acting in both hemopoietic cells and the BM microenvironment since embryogenesis. Large-scale application of NGS in these cases as well as in acquired CNP with a propensity to develop MDS/AML will significantly improve diagnosis and risk stratification. Longitudinal NGS studies will be performed to provide new insights into driver mutations and stepwise transformation. This WG will also investigate the role of cellular stress mechanisms in leukemic evolution. Novel tools, based on iPS cells and genome editing technologies will be developed to elucidate these stress mechanisms and interrogate the sequential events leading to MDS/AML. Key findings will be exploited to develop strategies to redirect premalignant progenitors back to normal. The WG will also establish ultra-deep sequencing of the critical region of the *CSF3R* and *RUNX1* genes and will also explore the importance of these acquired mutations in leukemic evolution through longitudinal retrospective analysis of multiple BM samples of large number of patients with Congenital Neutropenias. Animal models (Mice and Zebrafish) will be also used to study molecular mechanisms of leukemogenesis triggered by *CSF3R* and *RUNX1* mutations. The landscape and clonal dynamics of genome-wide somatic mutations associated with MDS/AML transformation in SDS will be investigated in single-cell derived HSC and progenitor cell colonies from these patients.

### Identification of targets for novel therapies

It has been shown that activation of the NAMPT/NAD^+^/SIRTs-mediated signaling as compensatory mechanisms of the G-CSF-mediated granulopoiesis results in induction of granulopoiesis in healthy individuals and CNP patients.^22^ This WG will promote studies on the evaluation of the effects of different NAMPT and SIRT1 activators as well as the NAMPT substrate, nicotinamide, on the G-CSF-induced granulopoiesis in vitro using iPS cell-based hemopoietic differentiation, primary BM HSCs, and in Zebrafish and xenograft mouse models. The WG will also facilitate the establishment of iPS cell lines from Congenital Neutropenia patients with different gene mutations and will study the effects of small molecules on the granulocytic differentiation. Small molecules capable of inducing differentiation will next be tested in primary HSCs and in animal models, including Zebrafish and Mice. We will also promote research on the potential of novel molecules to interfere with neutrophil elastase whose malfunction is a frequent cause of Congenital Neutropenia. Inhibitors of neutrophil elastase proteolytic activity are expected to broaden the therapeutic opportunities for these patients.^23^

### CNP patient Registries and biobanking

Partners from national CNP patient registries, biobanking consortia and SCNIR will contribute to the organization and interconnection of CNP patient Registries and Biobanks at the European level.^24^ The WG will contribute to the standardization of the modus operandi of the national CNP Registries across participant countries to ensure reliable statistics and standard indicators on incidence, prevalence, morbidity, mortality, infection rates, quality of life, proportion of patients receiving G-CSF and dose, number of patients undergoing HSC transplantation, comorbidities and rate of MDS/AML. Given that the availability of Biobanks is a prerequisite for progress in understanding CNPs and improving patient care, the WG will promote the systemic and standardized collection of patient samples and will propose guidelines for biological data collection, management, sharing and transfer according to the European Legal Framework taking also into account national and regional ethical and regulatory aspects. Issues specific to the type of samples (serum, blood and/or BM cells, others) and the time points of collection to allow pertinent study in close linkage with clinical data will be defined. Finally, the WG will propose a single classification of all different diseases associated with CNP.

## Capacity building within EuNet-INNOCHRON and beyond

The EuNet-INNOCHRON is an open network focused on the investigation, characterization, harmonization of diagnosis and treatment of CNPs, promotion of research, technology and entrepreneurial innovation, consisting so far of more than 30 countries. The Action was built on a network of experts in the field of CNP with long-standing collaborations and further enriched with scientists of diverse expertise to achieve an interdisciplinary and multidisciplinary approach to study the pathogenesis of CNP and is open to researchers, particularly at early career stages, related scientific societies, patient organizations, biotechnology and pharmaceutical companies. A key intention of EuNet-INNOCHRON is to foster broad collaborations and open the way for cutting-edge research and transfer of knowledge to the clinic. The consortium is anticipated to strengthen the interactions among experts in the field of CNP at the European level and beyond, to facilitate and extend the collaboration between existing outstanding networks such as EHA-Scientific Working Groups, SCNIR and national Registries for the introduction of common policies in CNP Registries and Biobanks, and, most importantly, to contribute to the generation of the future leading physicians and researchers in the field of CNPs with appropriate knowledge and skills to promote further research, innovation and personalized patient treatment.

## Authors’ Contribution

HAP conceptualized the manuscript. All authors contributed to the writing and editing of the manuscript. All authors approved the final version.

## Disclosures

HAP has participated in Advisory Boards for Novartis and Abbvie Companies; JP is chair of the Neutropenia Advisory Board at ApoPharma Ltd, Toronto, Canada, (Chieti Canada Ltd); CD has participated in Advisory Board for Novartis. The rest of the authors declare no conflicts of interest.

This article is based upon work from COST Action CA18233 “European Network for Innovative Diagnosis and treatment of Chronic Neutropenias, EuNet-INNOCHRON” (https://www.eunet-innochron.eu/) supported by COST (European Cooperation in Science and Technology).
